# Impact assessment of the European Clinical Trials Directive: a longitudinal, prospective, observational study analyzing patterns and trends in clinical drug trial applications submitted since 2001 to regulatory agencies in six EU countries

**DOI:** 10.1186/1745-6215-13-53

**Published:** 2012-04-29

**Authors:** Markus Hartmann

**Affiliations:** 1 European Consulting & Contracting in Oncology, St. Annastrasse 3, D-54295 Trier, Germany

## Abstract

**Background:**

Shifts in clinical trial application rates over time indicate if the attractiveness of a country or region for the conduct of clinical trials is growing or decreasing. The purpose of this observational study was to track changes in drug trial application patterns across several EU countries in order to analyze the medium-term impact of the EU Clinical Trials Directive 2001/20/EC on the conduct of drug trials.

**Methods:**

Rates of Clinical Trial Applications (CTA) for studies with medicinal products in those six countries in the EU, which authorize on average more than 500 trials per year, were analyzed. Publicly available figures on the number of annually submitted CTA, the distribution of trials per phase and the type of sponsorship were tracked; missing data were provided by national drug agencies.

**Results:**

Since 2001, the number of CTA in Italy and Spain increased significantly (5.0 and 2.5% average annual growth). For Italy, the gain was driven by a strong increase of applications from academic trial sponsors; Spain's growth was due to a rise in trials run by commercial sponsors. The Netherlands, Germany, France and the UK saw a decline (1.9, 2.3, 3.0 and 5.3% average annual diminution; significant (*P *< 0.05) except for Germany) in clinical drug trials. The decrease in the UK was caused by a sharp fall in academic trial activities. Across the six analyzed countries, no EU-wide trial-phase-specific patterns or trends were observed.

**Conclusions:**

The EU Clinical Trials Directive 2001/20/EC did not achieve the harmonization of clinical trial requirements across Europe. Rather, it resulted in the leveling of clinical trial activities caused by a continuing decrease in CTA rates in the Netherlands, Germany, France and the UK. Southern European countries, Italy and Spain, benefited to some extent from policy changes introduced by the Directive. In Italy's case, national funding measures helped to considerably promote the conduct of non-commercial trials. On the other hand, the EU Directive-driven transition from liberal policy environments, based on non-explicit trial approval through notifications, towards red-taped processes of trial authorization, contributed to the decreases in trial numbers in Germany and the UK. In the latter case, national research governance concerns had a share in the country's marked decline. However, different EU member states successfully developed best practices, which a new European legislation should take into consideration to resume Europe's attractiveness and international competitiveness for the conduct of clinical trials.

## Background

A strong clinical research sector and efficient trial infrastructures are deemed to be key requisites for a country's or region's capability to successfully contribute to the development of innovative health care products and to the evidence-based evaluation of the value and effectiveness of innovative as well as established products. The availability of sufficiently large pools of patients for inclusion in clinical trials, the rapidity of study set-up and recruitment of trial participants, the existence of a legal framework ensuring and promoting the GCP-like conduct of trials, and the general intention to keep the costs of any research endeavor as low as possible, constitute other commonly acknowledged elements for a country's or region's competitive capacity in a globalized clinical research landscape.

During the last decade, the true globalization of clinical research impacted tremendously on the perception of how clinical research would evolve further, raising some concerns in the United States about the future relevance of US-centered clinical drug research and the ethical and scientific implications of research globalization [1,2]. In Europe, the discussions on the continent's future role in clinical research focused most on the impact of the European Union Clinical Trials Directive (EUCTD) [3]. Intended to harmonize and simplify clinical trial requirements across the European Union (EU) and to sustain innovation and competitiveness, the Directive was widely bemoaned to raise legal obstacles, bureaucracy, work load and costs and to undermine Europe's position in clinical research [4]. Adopted in 2001, the Directive asked for implementation of its requirements until 1 May 2004 - the same day when 10 new member states from central, eastern and southern Europe entered the EU. Due to the complexity of the legal transposition process, many member states did not meet this deadline. Some states, among them France and the Netherlands, implemented the act with a two-year delay.

Especially, academic researchers continued to criticize the EUCTD - reference 4 provides a nice snapshot of the length and amplitude of the harsh discussions - and pushed, in line with pharmaceutical trade associations, the EU Commission to act. The Commission, the responsible policy maker in the field of clinical research, held a stakeholder conference investigating the operation of the EUCTD [5] in October 2007, in cooperation with the European Medicines Agency (EMA). After the conference and two comprehensive follow-up evaluations of the situation [6,7], the European Commission finally led a public consultation on the functioning of the Directive [8] and engaged, as an outcome of the consultation process [9], in a process to revise the legal framework for clinical drug research in the EU [10,11].

Despite several initiatives to analyze the situation of clinical drug research in the EU and to define priorities for future action, apart from single-country- or single-trial-based situation-analyses, only few overall data sets were available allowing to quantify the true impact of the EUCTD. One project (ICREL) provided an EU-wide situation analysis with focus on changes observed between 2003 and 2007 [6]. Given the aforementioned, the present study was initiated in 2006 to benchmark patterns and trends in the clinical research activities in Europe in the form of a *prior-posterior *analysis. Assuming that the detailed outcomes of the policy changes were to measure most adequately in those western European countries that had played for decades a leading role in clinical drug development data from these countries were retro- and prospectively collected. It was assumed that data from these core countries might serve as a surrogate for Europe's clinical research activity and competitiveness. Taking into account the lengthy implementation process of the EUCTD in several countries, a follow-up, including data until 2009, was deemed necessary to determine reliable pre- and post-implementation figures for comparison.

## Methods

Clinical trial applications (CTA) for studies with medicinal products in those six countries in the European Union (EU) (France, Germany, Italy, the Netherlands, Spain and the United Kingdom), which authorize on average more than 500 drug trials per year, were analyzed. The relatively large national drug agencies in these countries provide sufficiently detailed metrics to analyze overall patterns as well as specific variables over the whole observation period. The analysis covers the period from 2001, the year of publication of the EUCTD, until 2009. Publicly available figures from national drug agencies (performance metrics, annual reports and statistics) on the number of annually submitted CTA were tabulated; the distribution of trials per phase and sponsor-type was tracked as well. On request, national competent authorities (NCA) had added missing information, mainly with regard to the distribution of sponsorship (commercial sponsors (CS) versus non-commercial sponsors (NCS)) - a differentiation originating from the EUCTD and defined in the EU Commission's initial accompanying guidance [12] as follows: "A commercial sponsor is a person or organization that takes responsibility for a trial which at the time of the application is part of a development programme for a marketing authorisation of a medicinal product". Linear regression analysis was used to determine trends over time in the CTA patterns. Average annual growth rates (AAGR), a measure used to determine trends in the globalization of clinical trials [2,13], were calculated on the basis of the linear regression equation. It was assumed that the numerous factors impacting on a sponsor's decision to set-up a clinical research project and to submit a CTA for a given trial in one or several countries were leading to a quasi-linear course of applications over time. In this model, changes in a country's attractiveness for clinical research are displayed by the gradient.

In order to better take into account the individual country-specificities of the legal implementation process regarding the factors 'sponsorship' and 'trial phase', per-country arithmetic means of pre- and post-implementation CTA figures were determined on the basis of the number of applications in three consecutive years prior to and after the time point (or, in the case of France, time period), when the legal requirements changed. The significance of the impact was determined by the comparison of the means of trial application rates (unpaired *t*-test, assuming that variability of each sample is due to the sum of numerous independent factors). *P*-values ≤ 0.05 were considered to indicate significance. *P*-values ≤ 0.001 were considered to be highly significant. Data were analyzed with MedCalc Statistical Software Version 11.1.1.0 (MedCalc, Inc., Mariakerke, Belgium).

## Results

### Overall patterns

**Figure 1:**
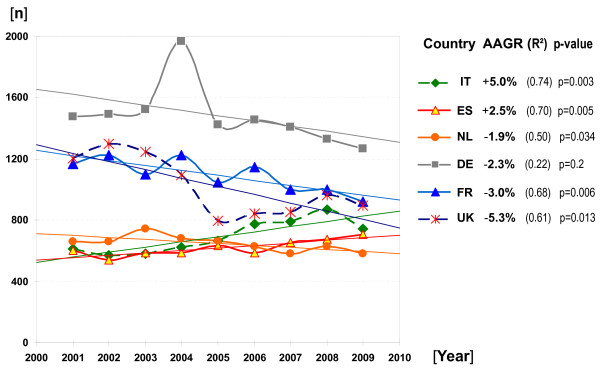
**Annual Clinical Trial Applications (CTA) and Average Annual Growth Rates (AAGR) for the period 2001 to 2009**. Figures for Germany comprise merged data for both national competent authorities (BfArM and the Paul-Ehrlich-Institute). Figures for the UK do not comprise phase I trials (often referred to in the UK as 'trials with healthy volunteers').

Over a period of nine years, a decrease in CTA rates was observed in the Netherlands, Germany, France and the United Kingdom, as determined by the AAGR (Figure 1). In Spain and Italy, the number of CTA was steadily growing. For Germany the delayed implementation of the EUCTD in August 2004 represented a major switch from a trial notification to a trial authorization system. A steep increase of old-style notifications was hence observed in the spring and early summer 2004. The resulting peak in applications depicts that many sponsors anticipated increased workload and costs introduced by the new legislation - a country-specific effect impacting on the reposted determination coefficient and statistical significance, too. Exploratory drug trials of minimal or non-interventional character were exempted from notification prior to August 2004; for this reason, the implementation of the EUCTD induced some bias in Germany's annual accounting for trial applications. It is estimated that trials with such 'minor interventional character', mostly conducted by practitioners and academic study groups, might have contributed to a 10 to 20% higher rate of total applications in the period 2001 to 2004. In the UK, trials with healthy volunteers, which include the vast majority of all phase I trials, were exempted from prior approval or notification prior to May 2004 [14]. Therefore, reported figures for the UK do not account for phase I trials.

**Figure 2:**
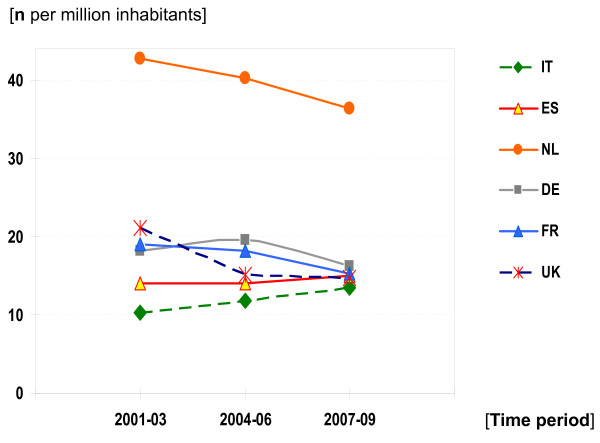
**Population-size-adjusted clinical trial activity across six EU member states**. The ultimate outcome of the EUCTD results in the leveling of clinical trials activities across the six analyzed EU member states. As in the previous figure, UK data do not include phase I trials.

In Figure 2, the population-size-adjusted clinical trial activity across the six EU member states over time is shown in terms of arithmetic means (numbers of initial CTA per country) for three consecutive years. The figure illustrates well that the ultimate outcome of the EUCTD results in the leveling of clinical trials activities across the six analyzed EU member states. Remarkable is the fact that the clinical trial density in the Netherlands is still much higher than in the other countries. The finding might be explained by the organization of the Dutch health care sector with a dominant role of hospitals for in-patient as well as for ambulatory care in line with the high population density and a traditionally open and positive attitude of the population and regulating authorities towards clinical drug research. This structural and behavioral vantage contributes also, in terms of the publication activity, to an internationally remarkable position of the Netherlands in clinical research: only the Scandinavian countries show higher *per-capita *publication rates [15].

### Impact of sponsorship

In Table 1, the findings regarding the impact of the factor 'sponsorship' are outlined. Only Italy showed a highly significant increase in non-commercial trial activities. In other countries, non-commercial trials either contributed less to the growth of trial activities than trials run by commercial sponsors (Spain), or were driving factors behind the observed general decline of applications (France, UK). Regarding both southern European countries, the comparison of means (Table 1, bottom) indicates that the growth in CTA in Italy was driven by non-commercial sponsors, whereas the observed growth in Spain was due to an increased activity of commercial sponsors. For the UK, the highly significant drop in trial activities just after the policy change confirms that the country's overall loss in trial applications over the last decade was predominantly caused by a decline of trial activities of non-commercial sponsors.

**Table 1:** Sponsorship and trial-phase specific patterns: AAGR (A) and *prior-posterior *implementation means (B)

	*All trials*	*CS trials*	*NCS trials*	*Ph I trials*	*Ph II trials*	*Ph III trials*	*Ph IV trials*
**A AAGR **(Period 2001 to 2009) (**%**)							
**IT**	+5.0*	+**1.3**	+13.0**	+32.8**	+6.3*	+**1.5**	+14.7**
**ES**	+2.5*	+**3.1**	+**0.7**	+**2.5**	+7.1*	-**1.1**	+**5.5**
**NL**	-1.9*	-2.0*	-**1.9**	+**1.1**	-3.8*	-2.8*	-5.7*
**DE**	-**2.3**	**--**	**--**	-5.3*	-**1.6**	-4.3*	+**6.4**
**FR**	-3.0*	-2.8*	-3.4*	-4.5*	-**0.3**	-**0.7**	-7.3**
**UK**	-5.3*	-**0.4**	-15.2*	**--**	**--**	**--**	**--**

**B Means **(*Prior-Posterior *Comparison) (**n CTA**)							
**IT**^**1**^	587 ⇨ 744*	**433 ⇨ 479**	153 ⇨ 260*	9 ⇨ 21*	206 ⇨ 280*	**311 ⇨ 344**	47 ⇨ 87*
**ES**^1^	**576 ⇨ 626**	**446 ⇨ 556**	**130 ⇨ 104**	**90 ⇨ 92**	**142 ⇨ 175**	**278 ⇨ 272**	**67 ⇨ 101**
**NL**^2^	697 ⇨ 597*	**405 ⇨ 351**	**292 ⇨ 246**	**139 ⇨ 126**	**161 ⇨ 132**	**203 ⇨ 177**	**73 ⇨ 67**
**DE**^1^	1,497 ⇨1,428*	(**1,300**) **⇨ 1,170**	(**197**) **⇨ 259**	462 ⇨ 340*	**340 ⇨ 321**	437 ⇨ 368*	65 ⇨ 144*
**FR**^3^	1,165 ⇨ 973*	885 ⇨ 727*	310 ⇨ 247*	288 ⇨ 221*	**291 ⇨ 288**	**365 ⇨ 350**	160 ⇨ 101*
**UK**^1,4^	1,248 ⇨ 832**	656 ⇨ 576**	592 ⇨ 256**	**nd ⇨ **(**301**)	**nd ⇨ 353**	**nd ⇨ 261**	**nd ⇨ 218**

In the case of Germany, exact distribution patterns for sponsorship could not been established for the period prior May 2004, the reported mean (n = 197) refers exclusively to studies initiated by universities or university hospitals. For the UK, trial phase specific distribution patterns prior to 2004 are unknown.

1: IT, ES, DE, UK: Means of three consecutive years prior to implementation (2001 to 2003) versus means for period posterior to implementation (2005 to 2007)

2: NL: Means of three consecutive years 'prior' (2003 to 2005) versus means for years 'posterior' to implementation (2007 to 2009)

3: FR: Means of three consecutive years 'prior' (2001 to 2003) versus years 'posterior' (2007 to 2009). Transition period for implementation of EUCTD in France: 2004 to 2006

4: UK: Means (All trials, CS trials, NCS trials) do not account for phase I trials ('trials with healthy volunteers')

CS/NCS, Commercial/Non-commercial Sponsor; nd, no data; *, significant, defined as *P *≤ 0.05; **, highly significant, defined as *P *≤ 0.001

### Trial phase specific patterns

All in all, no trial phase specific trends, interpretable as EU-wide exodus or influx of trials of a given phase or as intra-European shifts, were found (Table 1). In Germany and France, a significant cutback in phase I trial activities was observed. It is assumed that the cutback is even more accentuated in the UK, but due to the fact that the majority of phase I trials - those with 'healthy' volunteers - were exempted from notification and any regulatory supervision prior to May 2004, no data suitable for comparison were available. Remarkable is the fact that in Italy and Spain the proportion of phase I trials, compared to other trial phases, is still quite low - an observation which might be explained historically: by socio-cultural attitudes towards trials with no, or a very uncertain, direct therapeutic benefit for the participant on the one hand, and most notably by the absence of a relevant infrastructure of Phase I units on the other hand.

Numbers of phase II trials were augmented significantly in Spain and Italy; the latter being the only country reporting a slight increase in phase III trials as well. In contrast to this, the number of phase III trials in the Netherlands and Germany fell significantly. For the UK, detailed trial phase figures prior to 2004 were not tracked by the authorities; hence a *prior-posterior *analysis of means is not feasible.

Regarding phase IV trials, the observed highly significant decrease in France is noteworthy; for the Netherlands, as well, a decrease was noted. Due to the rising importance of phase IV trials in terms of post-approval market surveillance of newly approved as well as established medicines, rising rather than declining figures were expected, as documented indeed for Spain, Germany and, particularly, Italy.

As outlined before, 'Therapy Optimization Studies' of minor-interventional or non-interventional character were to some extent exempted prior to August 2004 from regulatory supervision, notably in Germany. The country's observed strong growth in phase IV trials since 2004 might be explained partly by this change in the regulatory policy. The concept and definition of what constitutes an interventional phase IV trial is still not harmonized across the EU. This might contribute to some bias in the accounting of phase IV CTA.

## Discussion

This paper provides a medium-term analysis of changes in clinical trial applications over time in six EU member states. An observation period of nine years (2001 to 2009) was deemed indispensable, to adequately track and compare application rates *prior *and *posterior *to the implementation of the EUCTD across Europe which took place between 2004 and 2006. As a snapshot in time, the results of the linear regression analysis do neither allow for extrapolation out of the observation period, nor any prevision about changes in CTA rates in other European countries. Similarly the reported *prior-posterior *means only allow for detection of trends and shifts within each country. Due to multiple applications in the case of international multi-center trials, any direct inter-country comparison of proportions or a summation of either means or trial approval numbers of all countries would be strongly biased and, therefore, misleading, especially for trial phases II, III and IV. Emphasis must be placed on the fact that in such *prior-posterior *studies one can never be certain to what degree unconsidered confounders are present - any interpretation of the presented data hence carries risks of making wrong interferences [16].

Regarding the main objective of this study, the assessment of the medium-term impact of the EUCTD on CTA rates in six relevant EU countries, several conclusions can be inferred from the above-presented results. First, over the last decade clinical drug research was not struggling in all countries covered by this analysis; the often bemoaned general negative effect [17,18] of the EUCTD on clinical drug research across Europe can not be generalized. The results indicate that the relative attractiveness of Italy and Spain for the conduct of trials has grown -commercial trial sponsors especially pay attention to factors such as trial set-up and approval times, improved GCP adherence or streamlined reporting requirements. Further analyses would be of interest to determine if other southern, but also eastern, EU countries also benefited from the implementation of the EUCTD.

Secondly, the results confirm that non-commercial trial sponsors reacted more sensitively to changes in the regulatory policy. The decline of the UK as well as the rise of Italy - the two most significant trends detected by this study - were both driven by striking shifts in the activity of academic trial sponsors. For non-commercial sponsors, costs and work-load related to the set-up and conduct of a trial were repeatedly reported to be determined for their capability to run clinical trials [19,20]. In this regard, the sponsor-specific variances in the CTA patterns are worth further discussion. Since 2003, Italy has applied a unique set of accompanying measures to foster clinical research [21]. In line with the implementation of the EUCTD, the specific needs of academic sponsors were encountered by an explicit recognition of the non-commercial sponsor statute in the national legislation, extra provisions for the cost takeover of Investigational Medicinal Products (IMP), as well as waivers of fees for the consultation of ethics committees and the Italian national competent authority. Particular alleviations for 'therapeutic strategy trials', everywhere a domain of investigator-driven research, have been laid down in Italy's legal decree on non-profit studies [22], an act unique in its kind in Europe [23]. In addition, a five percent levy on all promotional budgets of pharmaceutical companies was decreed to generate meaningful funding for independent drug research [24]. On the other hand, in 2009 another decree laid down minimum requirements for insurance policies which safeguard participants in clinical trials of medicinal products [25]. Asked to extend their existing insurance coverage to explicitly imply clinical trial activities or to set-up additional indemnity-covering insurances, many Italian institutions began to limit the number of trials initiated by their staff. As a consequence, in 2009, the number of non-commercial trials in Italy slumped for the first time after eight years of a consecutive rise.

This example shows that national policy-making is still highly influential on the conduct of trials. A second example concerns the UK, where the Department of Health, concerned by the observed strong decline of trial activities since 2003, began in 2006 to establish model clinical trial agreements. The department argued that such "nationally approved standard agreements help speed up the contracting process for clinical trials carried out in the NHS". The agreements are part of a system of reforms to streamline the process of conducting clinical trials in the National Health Service (NHS) [26]. It is assumed that the initiative, which was gratefully acknowledged by independent research organizations as well as by commercial sponsors, contributed to a certain extent to a temporary stabilization of CTA rates, as observed since 2006 (see Figure 1).

However, the situation in the UK remains a source of concern for clinical researchers as well as for national policy-makers. Findings from British investigators depict that, apart from the additional administrative burden introduced by the EUCTD in 2004, UK-specific artifacts in the trial authorization and supervision process continued to render clinicians' lives difficult [27]. The changes within the research ethics committees system produced a lot of insecurity, an over-interpretation of requirements, as well as a huge increase in required documentation. Indeed, local research facility scientific advisory committees had been set-up inside many regional trusts of the NHS resulting in a new multiplicity of ethics approvals. Different attitudes within the NHS were also observed in terms of research governance, as well as in indemnity and liability take-over, rendering the conduct of UK-wide multi-center studies quite complicated. These particularities caused the National Institute of Health Research (NIHR) in 2008 to establish a "bureaucracy busting mission", and the government to ask the Academy of Medical Sciences to thoroughly review the current regulatory and governance environment around research. Recommendations from this working group were published in early 2011 and may pay off in the coming years [28].

Many of the specific findings discussed above, such as the unprecedented decline of clinical research in the UK between 2002 and 2006 or the rise of phase IV trials in Germany, had been previously reported in the form of single observations [29,30]. On the other hand, only a few other publications analyzing medium- to long-term trends in clinical trial applications in Europe were published so far. A study similar in conception examined the effect of EUCTD on academic drug trials in Denmark, based on CTA figures spanning the period from 1993 until 2006 [31]. Overall figures from the EudraCT database, established in 2004 in order to set-up an EU-wide clinical trial register with a unique trial identifier for each new trial, were reported for the first time to the general public in the EU Commission's assessment paper from October 2009 [8]. The data point out that the absolute number of clinical drug trials applied for in the whole EU increased from 3,969 in 2005 to 5,028 in 2007. Since then the absolute number of trials began to decline; in 2009 4,491 new drug trials were initiated in the EU (in 2010: 4,193) [11]. During this period, approximately two-thirds of the trials were sponsored by the pharmaceutical industry. Neither the EU Commission nor the European Medicines Agency (EMA), which is in charge of the EudraCT database, had any comparative data on file for the period prior to May 2004.

In order to gain a clearer picture of the situation, the European Commission launched in 2008 a comprehensive study on the "Impact on Clinical Research of European Legislation" (ICREL) [6]. ICREL was a longitudinal, retrospective, observational and comparative study to assess the impact of the EUCTD "on the number, size, and nature of clinical trials, on workload, resources, costs and performance" [8]. For the project, comprehensive feedback was collected from trial sponsors, ethics committees and national competent authorities. The impact assessment was based on mean differences between 2003 and 2007, estimated to verify whether a marked change occurred over time. The project compiled a wealth of information on the EUCTD's outcomes [6], including feed-back from 25 national competent authorities in the EU. The total number of CTA per year during the observation period shifted from 8,022 (for 2003) to 9,363 (for 2007), revealing in accordance with the European Commission's data the ascendance of multinational trials in CTA rates: according to the Commission, approximately 25% of clinical trials in the EU are conducted in more than one country; these multinational trials account for approximately 60% of all CTA submitted in the EU [8]. Adding up all reported trial applications, the ICREL report concluded that the number of CTA between 2003 and 2007 did not change significantly with an observed slight increase of 1.5%. Respectively, a significant 10.5% increase for CTA submitted by commercial sponsors across the EU was reported, as well as a non-significant decrease of 25.6% in terms of CTA of non-commercial sponsors. The report noted also that the most "important clinical research countries experienced a certain decrease in their research activity due to a fall in the number of CTA sponsored by non-commercial entities". In contrast, "regulatory agencies with a small number of CTA tended to slightly increase their activity over time" [6].

Nevertheless, the ICREL data regarding CTA have two major limitations. One is the bias caused by the multiple accounting of CTA of multinational trials: true shifts in the absolute number of the largely multinational phase III trials, hence, impact more severely on overall figures than shifts in the absolute number of phase I trials. The second limitation relates to the fact that the CTA metrics in the report were reported in an anonymous manner, on request of some of the authorities which provided that data. The ICREL report, therefore, does not deliver information of how CTA rates were impacted at the national level.

The present results reported in this paper have a double significance. On the one hand, they provide a detailed first-ever situation analysis for those countries which are the heavyweights in clinical drug research in the EU. On the other hand, they represent a second, complementary analysis of CTA data, adding evidence to the understanding of the current situation in Europe and providing for the first time ever trial-phase specific metrics. At a glance, the data confirm ICREL's findings due to the fact that the source data are the same. Some differences in the magnitude of the described trends or in single findings are due to the differing observation periods and the chosen methods for data analysis. But both studies share the same main findings: (i) on the whole, CTA rates in Europe have been struggling for some time to show sustainable growth dynamics; (ii) non-commercial research in Europe is much more affected by the changed policy environment than commercial research; (iii) the decrease in non-commercial trial activities clearly affects growth rates in the leading EU countries, that is, Germany, France and the UK; and (iv) in some other countries, including Italy and Spain, the overall clinical research activity developed positively.

The presented trial phase specific patterns also match well with the first EudraCT-based data made public in February 2011 [11]. According to the European Commission, phase distribution pattern in terms of absolute numbers of new trials were unchanged between 2007 and 2010. As it can be expected for the existing, sequential R&D model, phase I trials were dominant (32.0% in average), followed by phase II (29.2%) and phase III trials (21.5%). 17.4% of all trials in the EU are interventional phase IV trials.

For a conclusive statement on the situation of clinical drug research in Europe, two questions must be answered. Are the observed discrepancies in growth among the six analyzed countries meaningful? Is the absence of growth in clinical research in Europe part of a global trend, or a regional phenomenon?

Surely discrepancies in growth are meaningful for patients, clinical researchers and health care systems in each country, as clinical research is an essential component of a high quality healthcare system [32]. Growth is linked to improved and faster access to new, promising therapies, the contribution to evidence-building in medicine, and to investments as well as the capacity-building in the healthcare research sector. The reported intra-European trends are meaningful not only from the research perspective, but also from the public health perspective: population-size-adjusted trial activity can be seen as a surrogate indicator in terms of access to innovative medicines and state-of-the-art medical care. In this respect, the observed shifts among countries result in some leveling of clinical research activities. Italy, prior to 2004 was still the taillight in terms of numbers of initiated trials per year and per million inhabitants (10.1 trials/year/million inhabitants), but has now closed the gap with its peers (Figure 2). Despite some methodological limitations and due to the lack of quantifiable variables, the relative change of CTA over time per country, expressed in terms of AAGR can be considered as a valid benchmark for the present analysis. Nevertheless, caution is required in the interpretation of the presented data, as the described outcomes are determined by complex multifactor processes.

Concerning the global situation, it was repeatedly reported that a world-wide shift in clinical trials to so-called emerging regions is under way [2,33]. The European Commission in its initial assessment report, as well as the European Medicines Agency came to similar conclusions, stating that in addition to North America, particularly Asia and Latin America began to recruit meaningful numbers of patients in international trials used for marketing authorization applications in the EU [8,34]. Available comparative figures from national authorities and presentations of authority officials held at various symposia reveal that the CTA rates in North America and Asia over the last decade grew on average more than 5% per annum; that is to say more than in Europe [35]. Japan, itself facing problems with its clinical research competitiveness, has reported a 4% annual growth for the period between 2001 and 2009, doing nearly as well as Italy. Average annual growth rates between 5 and 10% were found for North America (USA 7%, Canada 8%) and for several south-eastern Asian countries (Taiwan 6%, Malaysia 6%, Singapore 7%). Other Asian countries, such as South Korea and China, who adapted rather late to international trial standards and GCP and who just began a few years ago to get involved into the global drug development process [36], currently experience annual growth rates of 30% and more. At a glance these comparative data underline that the growth rates in Europe, especially the observed decline in north-western Europe, are underpinning the continent's fall in clinical drug research.

## Conclusions

In conclusion, the implementation of the EUCTD did not result in the intended harmonization of clinical trial requirements across the six analyzed EU member states but in the leveling of clinical trial activities. The expected increase of clinical research activity aimed by the EUCTD has not occurred in Europe. In contrast, the number of trials decreased in central and north-west Europe. Policy-makers in the EU should take notice of the lack of growth in a sector crucial for future biomedical competitiveness.

The process and the elements of the implementation of the EUCTD across Europe impacted positively or negatively on the overall outcome in respect to CTA rates. In Italy's case, national measures had a synergistic effect and promoted the conduct of trials. In addition to reduced trial approval timelines through streamlined and more transparent processes as introduced by the EUCTD - Italy as well as Spain benefited from this factor - the Italian measures supported non-commercial research through adequate new funding and research governance provisions. The additional burden imposed by the Directive deprived the UK of its former regulatory simplicity - especially for Phase I trials with healthy volunteers. This negative effect has been accentuated by structural problems inherent to its national health care research governance system.

Different EU member states have successfully developed best practices. In light of the reported outcomes, it is hoped that the European national policy-makers are exchanging their best practices gained so far. The intended revision of the EUCTD bears the promises to overcome the principle obstacles identified during the last five years, to affirm Europe's role in clinical research and to rebuild sponsors' and trialists' engagement in clinical drug research in Europe.

## Abbreviations

AAGR: Average annual growth rates; CS: Commercial sponsors; CTA: Clinical trial applications; DE: Germany; ES: Spain; EU: European Union; EUCTD: European Union Clinical trials Directive; FR: France; GCP: Good Clinical Practice; ICREL: Impact on Clinical Research of European Legislation; IT: Italy; NCS: Non-commercial sponsors; nd: no data; NHS: National Health Service; NL: The Netherlands; UK: United Kingdom.

## Competing interests

The author declares that they have no competing interests.

## Authors' information

The author works as an independent medical and regulatory affairs consultant. Focusing on regulatory aspects of clinical research, the presented data dealing with policy outcomes research are based on a master thesis, submitted in autumn 2005 to the University of Bonn, Germany [37]. For the master thesis and this follow-up project, neither public nor private (third-party) funding was available/obtained.

## Supplementary Material

Additional file 1 **Directory of data sources**. Click here for file
