# Supplementary material for: Impact assessment of the European Clinical Trials Directive: a longitudinal, prospective, observational study analyzing patterns and trends in clinical drug trial applications submitted since 2001 to regulatory agencies in six EU countries
Source: Trials. 2012 Apr 29;13:53. doi: 10.1186/1745-6215-13-53 (PMC3349611; doi:10.1186/1745-6215-13-53)
Supplement: Additional file 1 — Directory of data sources. [file 1745-6215-13-53-S1.PDF]

Additional file

Directory of data sources
**Italy**: **Agenzia Italiano del Farmaco (AIFA) / Osservatorio Nazionale sulle Sperimentazioni Cliniche (OsSC)**
Annuale Rapporto Nazionale (3° - 9° Rapporto, 2004-2009) http://ricerca-clinica.agenziafarmaco.it/it/node/482
**Spain**: **Agencia Española de Medicamentos y Productos Sanitarios (AEMPS)**
AEMPS Memorias (2001-02, 2003-04, 2005-06, 2007, 2008, 2009) http://www.aemps.es/actividad/nosotros/memoria/home.htm
**Netherlands**: CBG-MEB / **Centrale Commissie Mensgebonden Onderzoek (CCMO)**
CCMO Jaarverslag 2001-1009 http://www.ccmo-online.nl/main.asp?pid=2&sid=5
**Germany**: **Bundesinstitut für Arzneimittel und Medizinprodukte (BfArM) & Paul-Ehrlich-Institut (PEI)**
BfArM-Statistiken Klinische Prüfung 2005-09 (earlier versions covering years 2000-04 on file, not available any longer on BfArM's website) http://www.bfarm.de/cln_103/DE/Arzneimittel/1_vorDerZul/klinPr/klin_prf_genehm/Statistik.html?nn=1015162 (Additional data on trial sponsorship patterns were provided on request in 2007, 2009 and 2010 by Th. Sudhop, J. Schriever and K. Mallinckrodt-Pape (all BfArM))<br>PEI Bearbeitungsstatistiken (2004-09) http://www.pei.de/cln_092/nn_160648/DE/infos/pu/02-klinische-pruefung/klin-pruef-statistik/klin-pruef-statistik-node.html?__nnn=true (Additional data for years 2001-04 were made public by H. Krafft (PEI) at ZAFES-Symposium 2007)
**France**: **Agence française de sécurité sanitaire des produits de santé (Afssaps)**
Afssaps Rapports Annuels 2001-09 : http://www.afssaps.fr/Afssaps-media/Publications/Bilans-Rapports-d-activite-Afssaps-publications-institutionnelles (Additional data for years 2001-2004 were provided on request by Afssaps in 2007 by C. Belorgey and S. Touatier)
**UK**: **Medicines and Healthcare products Regulatory Agency (MHRA)**
MHRA Annual Reports 2001-09 http://www.mhra.gov.uk/Publications/Corporate/AnnualReports/index.htm; MHRA Clinical Trial Metrics http://www.mhra.gov.uk/Howweregulate/Medicines/Licensingofmedicines/Clinicaltrials/UKclinicaltrialauthorisationassessmentperformance/index.htm (Calendar-year adjusted (additional) data for years 2001-2004 were provided on request by HHRA in 2009 by M. Ward)
**USA**: **U.S. Food and Drug Administration (FDA) - Centre for Drug Evaluation Research (CDER) ***
CDER Original INDs received (1986-2009) http://www.fda.gov/downloads/Drugs/DevelopmentApprovalProcess/HowDrugsareDevelopedandApproved/DrugandBiologicApprovalReports/UCM165257.pdf
**Canada**: **Health Canada – Therapeutic Products Directorate (TPD)**
TPD Annual Reports: http://www.hc-sc.gc.ca/dhp-mps/prodpharma/applic-demande/docs/perform-rendement/ar-ra/index-eng.php
**Japan**: **Pharmaceuticals and Medical Devices Agency (PMDA)**
PMDA Annual Reports http://www.pmda.go.jp/english/about/annual.html and presentations from PMDA officials: H. Chimura, 9th Kitasato University – Harvard School of Public Health Symposium, Tokyo 2009 http://www.pharm.kitasato-u.ac.jp/biostatis/khsympo200909/doc/chimura.pdf; S. Toyoshima, DIA 22nd Annual EuroMeeting, Monaco 2010 (data on file).
**Taiwan**: **Department of Health**
Presentation T. Tanako, APEC Clinical Trial Workshop, Seoul 2010 http://www.apec-ahc.org/files/tp201002/Session4_TetsuomiTakano.pdf
**Malaysia**: **National Pharmaceutical Control Bureau (NPCB) / Clinical Research Center (CRC)**
NPCB Annual Reports http://portal.bpfk.gov.my/index.cfm?menuid=25&parentid=4, CRC news: http://www.crc.gov.my/ and presentations from CRC Officials: T.-O. Lim, Clinical Trial Magnifier Conference, Hong Kong 2009 (data on file)
**Singapore**: **Health Sciences Authority (HSA)**
HSA Clinical Trial Statistics: http://www.hsa.gov.sg/publish/hsaportal/en/health_products_regulation/clinical_trials/statistics.html
**South Korea**: **Korean FDA (KFDA) / Korean Network Clinical Trials (KoNECT)**
KFDA: http://www.kfda.go.kr/index.jsp and http://www.konect.or.kr/. Presentations from KoNECT officials: D. Lee, Clinical Trial Magnifier Conference, Hong Kong 2009. KoNECT news 2010 (data on file)
**China**: **State Food and Drug Agency (SFDA)**
SFDA: http://www.sfda.gov.cn/WS01/CL0001/ and presentations from SFDA officials: : C. Xiaoyuan, 8th Kitasato University – Harvard School of Public Health Symposium, Tokyo 2008 http://www.pharm.kitasato-u.ac.jp/biostatis/khsympo200809/doc/chen.pdf

* Respective statistics from CBER who regulates biological products in the USA (blood products, vaccines, complex biotechnology products, etc) are not available
